# Voting-Based Cancer Module Identification by Combining Topological and Data-Driven Properties

**DOI:** 10.1371/journal.pone.0070498

**Published:** 2013-08-05

**Authors:** A. K. M. Azad, Hyunju Lee

**Affiliations:** School of Information and Communications, Gwangju Institute of Science and Technology, Gwangju, South Korea; Wayne State University, United States of America

## Abstract

Recently, computational approaches integrating copy number aberrations (CNAs) and gene expression (GE) have been extensively studied to identify cancer-related genes and pathways. In this work, we integrate these two data sets with protein-protein interaction (PPI) information to find cancer-related functional modules. To integrate CNA and GE data, we first built a gene-gene relationship network from a set of seed genes by enumerating all types of pairwise correlations, e.g. GE-GE, CNA-GE, and CNA-CNA, over multiple patients. Next, we propose a voting-based cancer module identification algorithm by combining topological and data-driven properties (VToD algorithm) by using the gene-gene relationship network as a source of data-driven information, and the PPI data as topological information. We applied the VToD algorithm to 266 glioblastoma multiforme (GBM) and 96 ovarian carcinoma (OVC) samples that have both expression and copy number measurements, and identified 22 GBM modules and 23 OVC modules. Among 22 GBM modules, 15, 12, and 20 modules were significantly enriched with cancer-related KEGG, BioCarta pathways, and GO terms, respectively. Among 23 OVC modules, 19, 18, and 23 modules were significantly enriched with cancer-related KEGG, BioCarta pathways, and GO terms, respectively. Similarly, we also observed that 9 and 2 GBM modules and 15 and 18 OVC modules were enriched with cancer gene census (CGC) and specific cancer driver genes, respectively. Our proposed module-detection algorithm significantly outperformed other existing methods in terms of both functional and cancer gene set enrichments. Most of the cancer-related pathways from both cancer data sets found in our algorithm contained more than two types of gene-gene relationships, showing strong positive correlations between the number of different types of relationship and CGC enrichment 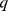-values (0.64 for GBM and 0.49 for OVC). This study suggests that identified modules containing both expression changes and CNAs can explain cancer-related activities with greater insights.

## Introduction

Cancer is a common genetic disease and a worldwide leading cause of death. Cancer genomics identifies changes of genes that play important roles in cancer initiation and progression. Decades of research have revealed that cancer is closely related to abnormal changes in regulatory and signaling pathways during its growth and malignance [1], [2]; such dysregulations in key pathways occur due to combinations of genetic alterations and expression changes of oncogenes or tumor suppressor genes [3]–[5]. Therefore, many algorithms have been developed to identify pathways related to cancer [6]–[9] using DNA CNAs, GE changes, PPIs, and so on.

Extensive uses of GE for studying molecular pathways have helped in classifying cancer subtypes, predicting prognosis, and developing drugs for cancer. However, using only GE data for identifying cancer-related genes is not enough because some important genes in cancer-related pathways might not be differentially expressed and some differentially expressed genes might not be relevant to cancer. CNAs are structural variations of DNA sequences that represent abnormal copies of DNA segments in a form of deletion or amplification in the cell [10]. CNAs are known to be a hallmark of cancer, and methods including GISTIC [11], RAE [12], and WIFA [13] have been used to detect cancer-driver genes in aberrant genomic regions. A recent large-scale analysis of GBM samples from The Cancer Genome Atlas (TCGA) [8] showed genetic alterations including mutations, deletions, and amplifications of DNA in 78%, 87%, and 88% of 206 GBM samples in the core components of RB, TP53, and RTK/PI3K pathways, respectively.

Several studies have recently reported the importance of integrating CNAs and GE data sets for the identification of cancer-related pathways. TCGA research on ovarian cancer showed that genetic alterations and gene expression changes simultaneously occur in the retinoblastoma signaling pathway [14]. Jörnsten *et al.* [15] developed a model that explains the effects of CNAs on GE in a large-scale network. Based on the model, prognostic scores were calculated and cancer-related genes were identified. Akavia *et al.* [16] employed an integrative Bayesian approach to identify biologically and therapeutically important driver genes in genetically altered regions by associating candidate driver genes with differentially expressed genes. They applied the proposed method to a melanoma data set and identified known driver genes in melanoma, along with novel cancer driver genes TBC1D16 and RAB27A. An important progress in combining CNAs and GE is analyzing genes as a module rather than as individual genes. Witten *et al.* [17] applied canonical correlation analysis for integrating CNAs and GE. This method links CNA modules with GE modules and optimizes CNA-GE interactions.

In constructing modules or subnetworks, PPIs have been used as prior information to incorporate connectivity among genes. Cerami *et al.* [9] proposed a method to construct subnetworks containing a significant number of mutated genes using human PPIs and to identify pathways that are related to GBM. Chuang *et al.* [6] proposed an approach of integrating PPIs and GE data sets to identify subnetwork markers that classify metastatic and non-metastatic tumors.

We propose a computational framework to incorporate CNA-CNA, CNA-GE, and GE-GE relationships to protein interaction network to identify cancer-related modules in which genetic changes of genes are explained by these relationships. Although the GE-GE relationship has been studied for decades [18]–[20], CNA-CNA [21]–[23] and CNA-GE [7], [24]–[27] relationships have only been recently studied. It is observed that amplifications and deletions of DNA segments can affect expression levels of genes in the same location, as well as distantly located genes [25]. This trans-located association between CNA and GE can be one of the mechanisms explaining complicated relationships between genes in the signaling and regulatory pathways. To incorporate these complex relationships, we construct a gene-gene relationship network using differentially expressed and significantly copy number altered genes in paired data sets containing both DNA and RNA data on the same set of patients. Then, we also incorporate PPI information to exploit prior functional dependencies between genes. We used a voting approach to find representative genes that are strongly related to other genes through associations among CNAs, GE, and PPIs. These representative genes are used to construct pre-modules by including strongly related genes. Then, pre-modules are merged with other pre-modules that have statistically significant associations through CNAs, GE, and PPI relationships, and final modules are generated.

The proposed approach was applied to GE and CNAs data of GBM and OVC samples from TCGA to identify cancer-related modules. The identified modules were assessed in two aspects: their functional coherence and relevance to cancer. To test that the modules are composed of functionally coherent genes, we applied functional enrichment tests using KEGG [28], BioCarta pathways [29], and GO biological process [30]. To test that the generated modules are related to cancer, we first selected cancer-related pathways from these three categories of pathways. Since there is still no consensus about which pathways or functional terms are related to cancer, we consider that a pathway is related to cancer if it is significantly enriched with cancer-related genes from a cancer gene census (CGC) [31]. Then, we applied enrichment tests using these cancer-related pathways. Our results showed that cancer-related pathways were enriched with our identified modules in both GBM and OVC data sets, and that a significant number of genes in the modules were associated with others through CNA-CNA, CNA-GE, and GE-GE relationships.

## Results

### A Framework for Combining Topological and Data-driven Properties

We developed the VToD approach to construct modules that are composed of a set of functionally coherent and cancer-related genes. VToD was developed based on four main ideas; (i) genes with similar gene expression profiles and copy number changes are more likely to be in the same module, (ii) genes can be assigned into multiple modules to reflect the biological knowledge that some genes are involved in multiple pathways, (iii) genes in a short distance in the PPI network are more likely to belong to the same module, and (iv) hub genes in the PPI network are more likely to be included in the modules since many hub genes having a large number of interacting partners may contribute to cancer development. The former two ideas consider data-driven properties and the latter two reflect topological properties of genes within the PPI network.

**Figure 1:**
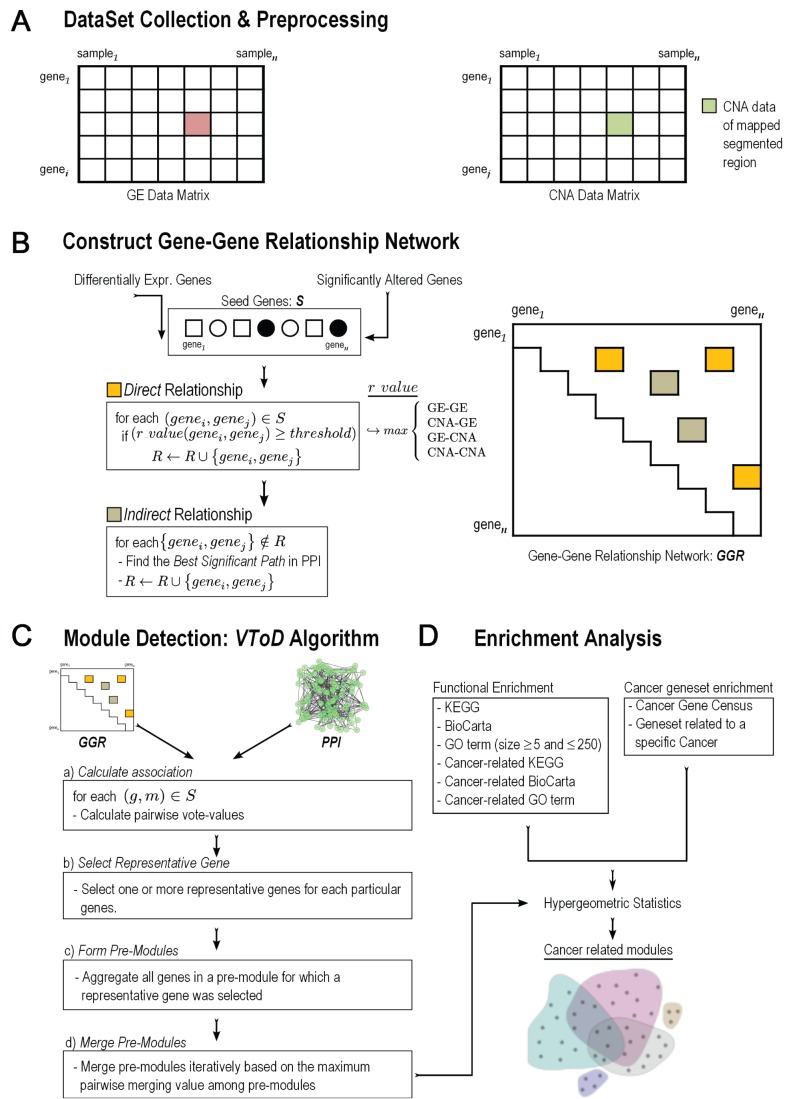
A schematic of our approach. (A) Gene expressions and their paired CNA data are collected. (B) A gene-gene relationship network, *GGR*, is constructed using direct and indirect relationships of GE-GE, CNA-GE, and CNA-CNA. (C) A novel algorithm, VToD, finds overlapping modules combining the *GGR* network and PPI information. (D) Functional and cancer gene set enrichments are tested for identified modules.

The schematic diagram of our proposed VToD method is shown in Figure 1. VToD constructs a gene-gene relationship network, 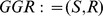 by integrating GE and CNA data sets, where 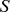 is a set of seed genes and 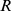 is a set of gene-gene relationships. Seed genes are selected by combining differentially expressed (DE) genes and CNA genes, where CNA genes are obtained from TCGA [8], [14] and listed in Table S1. For GBM, 4,821 seed genes were selected by combining 2,976 DE genes and 2,073 CNA genes. For OVC, 6,649 seed genes were constructed by 710 DE genes and 6,510 CNA genes. Note that some seed genes are both differentially expressed and copy number altered. The gene-gene relationships 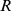 were constructed, where two genes have strong correlation in at least one of three types of relationships: GE-GE, CNA-GE, and CNA-CNA. Then, VToD integrates a PPI data set with the gene-gene relationship network *GGR* by following four major steps.

1. *Calculate the association between genes:* For every two genes 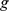 and 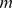, an association value from gene 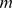 to gene 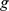 is calculated by combining the gene-gene relationship and the PPI data set. The association value is called a 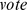-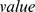 in this study, since we assume that gene 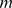 votes for gene 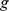 to represent the strength of the association between two genes.
2. *Select representative genes of each gene:* For gene 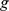, vote-values from all other genes are sorted in descending order, and genes located within the top 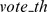
% of the vote-values are selected as the representative genes of the gene 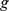.
3. *Form pre-modules:* If a gene 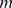 is selected as a representative gene from multiple genes, other genes selecting the gene 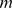 as the representative gene along with the gene 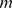 itself form a pre-module.
4. *Merge pre-modules:* Two pre-modules are merged if pairwise members of the two pre-modules are highly related in the gene-gene relationship network and are closely connected in the PPI network.

The VToD algorithm is inspired by a dynamic signal transduction system (STM) algorithm [32] in which, for each gene, the most associated genes are selected to form pre-modules based on the PPI topology only. However, the clear distinction lies between STM and VToD in the process of (i) calculating the association between two genes and (ii) merging pre-modules, since our approach integrates GE, CNAs, and PPI data sets.

The constructed modules were assessed in two aspects; (i) we measured functional relevance of the identified modules by testing whether genes in a module were enriched for KEGG, BioCarta pathways, and biological processes in GO terms (called a functional enrichment test), and (ii) we assessed the relevance of the modules to cancer by applying an enrichment test to the cancer-related pathways or cancer-related biological functions, which are subsets of the above three categories of pathways/GO terms enriched with cancer-related genes from CGC [31] (called a cancer-related pathway enrichment test). Further, we tested whether genes in the identified modules were enriched with cancer genes from CGC, GBM driver genes [33], and OVC-related genes [34]. In these assessments, the hypergeometric statistics were used for the enrichment test.

### Modules from the VToD Algorithm

**Figure 2:**
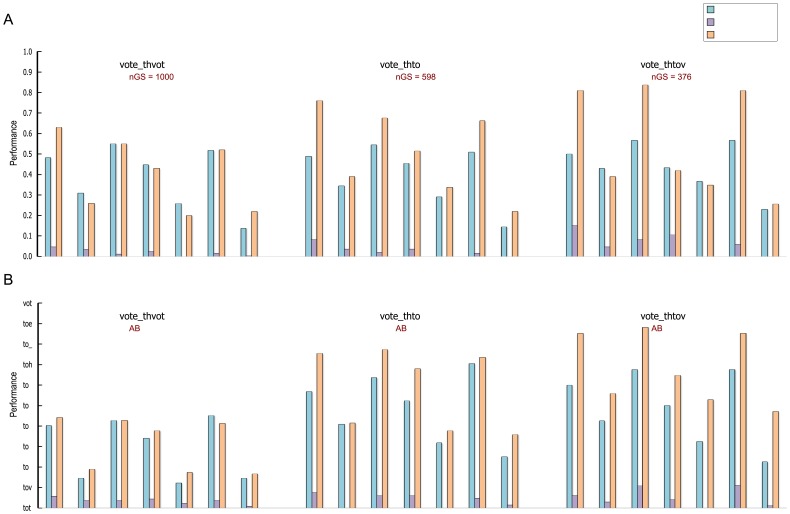
Comparative functional enrichments of pre-modules generated using different vote thresholds. (A) is for GBM and (B) is for OVC. Bars represent fractions of modules enriched with KEGG, BioCarta, GO biological process, cancer-related KEGG, cancer-related BioCarta, cancer-related GO biological process, and cancer gene census (CGC) for three different vote thresholds. Additionally, in each case, vote-values were computed using only topological properties, using only data-driven properties, and by combining them to compare their individual effects on performance. The numbers of genes (nGS) in each pre-module set are shown correspondingly.

The distributions of all enumerated pairwise gene-gene relationships (GE-GE, CNA-GE, and CNA-CNA) among seed genes are shown in Figure S1, and the distributions of all vote-values for GBM and OVC data sets are shown in Figure S2. Since the number of pre-modules depends on the 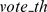
% values in Step 2 of the VToD algorithm, we tried three 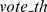 values to examine how 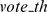 values affect on the constructed pre-modules. Vote-values of the top 1%, 0.25%, and 0.1% eventually yielded 100, 68, and 43 pre-modules for GBM, and 138, 53, and 34 pre-modules for OVC. Then, we applied the functional enrichment tests and cancer-related pathway enrichment tests to pre-modules generated using the three threshold values above. Figure 2 shows the fraction of enriched pre-modules; although many pre-modules have significant overlaps with known pathways across all three thresholds, pre-modules from 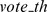  = 0.25% and 0.1% have more overlaps with the pathways compared to 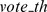  = 1%, showing that higher vote-values generate higher fraction of functionally relevant and cancer-related modules.

We also tested the importance of considering both topological and data-driven properties for pairwise vote calculation. We generated pre-modules using only topological and only data-driven properties. When the single property was used, the same number of gene pairs was selected with that of gene pairs selected by combining both properties for each value of 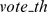. Across all three values for the 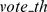 threshold, the fraction of functionally enriched modules was higher when topological and data-driven properties were combined than when only a single property was used for both GBM and OVC, as shown in Figure 2.

We chose 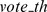  = 0.1% as a threshold for further analysis. Using this threshold, for GBM, 43 pre-modules were obtained. By merging these pre-modules, 22 modules were generated, and the average number of genes in the modules was 24. For OVC, using the same threshold, 34 pre-modules were generated, and 23 modules were obtained after merging pre-modules, where the average of number of genes is 57. All genes in the modules are listed in Table S2 and Table S3. The statistical significance of the identified modules is shown in Figure S3.

Since the VToD algorithm allows multiple appearances of genes in several modules, we calculated the average ratio of common genes between modules. For GBM, the ratio of common gene was 16.07%, which was similar to those of the KEGG and BioCarta pathways. Also, the distribution of ratios of common genes was calculated. Around half of the modules had 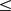 10% of common genes, which indicates that final modules will be enriched with distinct functional pathways or terms (Figures S4A and S4B). We also investigated three different types of direct relationships (GE-GE, CNA-GE, and CNA-CNA) between the gene pairs within each of these 22 GBM modules (Figure S5A). Around 64% of the modules contained at least two types of relationships, showing (i) that genes with similar gene expression and DNA copy number changes are more likely to be in the same module, and (ii) that the activity of the genes in these identified modules can be explained by different molecular mechanisms (Table S4).

For 23 OVC modules, the average ratio of common genes was 11.68%, which was also lower than those from KEGG and BioCarta, and more than half of the 23 OVC modules had 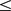 10% of common genes (Figures S4C and S4D). Around 83% of all 23 OVC modules (Figure S5B) contained at least two types of direct relationships.

#### Cancer-related modules identified by the VToD algorithm for GBM

We applied functional and cancer gene set enrichment tests to 22 GBM modules. We found that 19 (86.36%), 14 (63.63%), and 20 (90.9%) modules were significantly enriched (FDR 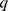-value 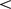 0.05) with at least one KEGG, BioCarta, or GO terms, respectively, showing that identified modules are functionally coherent. Also, 15 (68.18%), 12 (54.55%), and 20 (90.9%) GBM modules were significantly enriched with cancer-related KEGG, BioCarta pathways, and GO terms, respectively. In the case of the cancer gene set enrichment test, 9 and 2 GBM modules had significant overlap (FDR 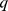-value 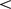 0.05) with CGC [31] and GBM-related genes [33], respectively. These results show that our modules are related to cancer development. Table 1 shows the summary of the top five selected modules ordered by GBM-related gene enrichment 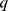-values; these modules contain many GBM-related genes. All enrichment results for the GBM data set are shown in Tables S4, S5, S6, and S7.

**Table 1:** Summary of functional and cancer gene set enrichments for selected GBM modules (sorted by driver gene set enrichment).

Module ID(Size)	# of enrichedpathways *^a,b,c^*& cancer-relatedpathways*^d,e,f^*	% of gene-gene directrelations*^x,y,z^*	# of CGC & GBMgenes ( 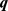 -values)	Enriched cancer genes in modules ^‡^
12	31, 40, 51	26.67%	4 (9.15 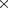 10 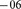 )	***EGFR***,***RB1***,SMAD4,
(10)	&	6.67%	3 (2.0 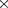 10 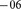 )	***TP53***
	34, 37, 57	6.67%		
2	37, 49, 73	26.59%	10 (1.05 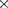 10 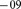 )	***TP53***,BRCA1,BRCA2,
(48)	&	0.79%	2 (1.02 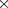 10 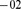 )	DDX5,MDM2,MDM4,NPM1,
	40, 48, 92	0.71%		DAXX,***TEP1***,WRN
17	29, 54, 26	41.82%	3 (4.98 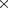 10 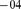 )	JAK2,***EGFR***,RAF1
(11)	&	3.64%	1 (5.61 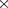 10 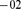 )	
	37, 52, 38	1.82%		
8	30, 39, 42	30.64%	6 (4.95 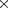 10 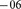 )	***EGFR***,CBLC,FAS,JAK2,
(33)	&	1.33%	1 (1.32 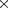 10 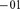 )	MET,MYC
	34, 37, 52	3.79%		
1	30, 49, 21	24.51%	6 (6.91 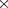 10 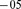 )	APC,BRAF,***EGFR***,
(55)	&	1.62%	1 (1.8 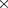 10 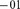 )	PPP2R1A,RAF1,WT1
	37, 37, 24	0.54%		

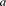
KEGG,

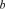
BioCarta,

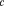
GO Term;

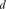
cancer-related subset of KEGG,

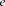
cancer-related subset of BioCarta,

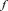
cancer-related subset of GO Term;

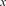
GE-GE,

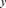
CNA-GE,

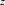
CNA-CNA relationships;

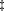
Gene symbols in bold text are GBM-related genes; the remainings are CGC genes.

**Figure 3:**
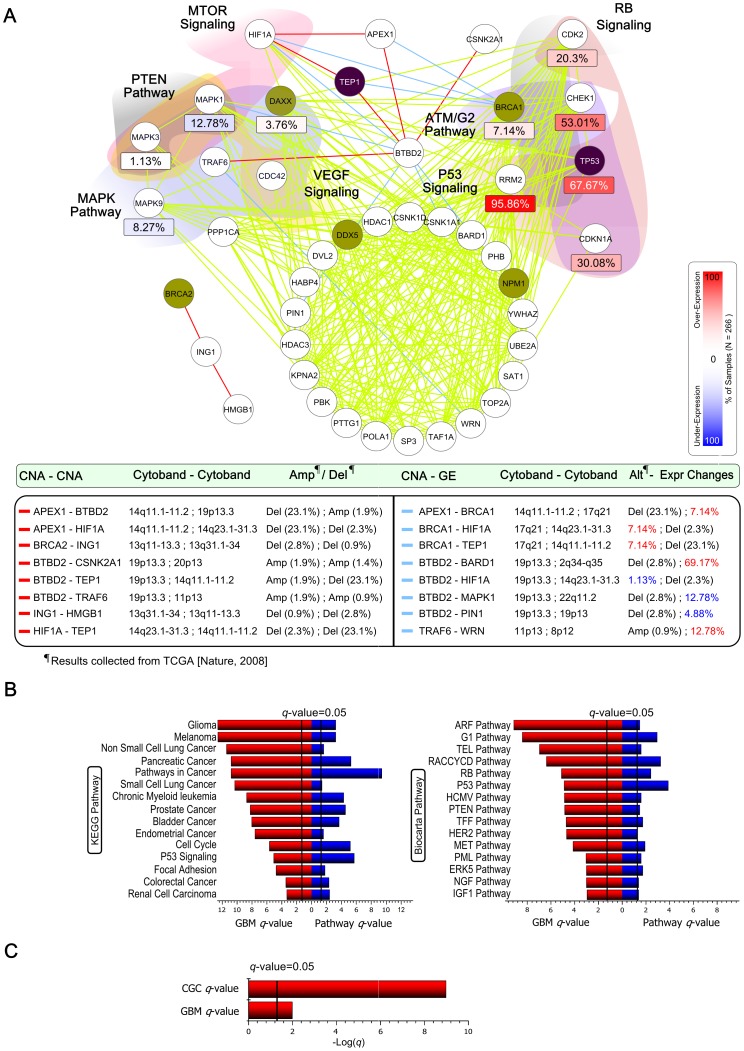
Analysis of GBM Module 2. (A) A network view of GBM Module 2 using only direct relationships, drawn by Cytoscape [70]. Genes were grouped together based on the overlap with BioCarta pathways, and the percentages of samples with CNAs and GE changes are shown. CGC genes are colored in olive and GBM genes are in purple. Cytoband and Amp/Del (or Alteration-Expression Changes) information for CNA-CNA (or CNA-GE) pairs are shown in the inset table. (B) Pathway enrichment tests with KEGG and BioCarta pathways for this module are shown. Blue bars indicate the enrichment 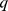-values of pathways and red bars indicate the overlap 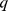-values between the pathway and GBM driver genes. Black vertical bars show 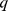-value threshold, 0.05, and the width of the horizontal bars depends on 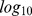
(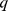-value). (C) Red bars show the overlapping 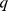-value with CGC and GBM driver genes.

We selected GBM Module 2 to explain in detail how genes are interacting with other genes and are involved in biological pathways in modules. We selected this module for further explanation since it has a low enrichment 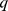-value with cancer gene sets, and contains gene pairs with strong correlations in three types of direct relationships. This module contains 1,080 gene pairs from 48 genes, and among them there were 300 GE-GE, 9 CNA-GE, and 8 CNA-CNA direct relationships. Figure 3A shows the network view of the GBM Module 2 with direct relationships only. There were three types of edges in this network: i) red edges for CNA-CNA, ii) blue edges for CNA-GE, and iii) green edges for GE-GE relationships between two genes. Genes belonging to significantly enriched pathways/terms were grouped together. Information for DNA CNAs and/or expression changes for genes were also labeled with them within each group. Frequencies of copy number changes were presented as a percentage of 206 GBM samples with either focal amplification or homozygous deletion in [8]. To count the fraction of tumor samples with gene expression changes for 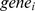, we considered that a tumor sample 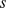 is over- or under-expressed if the value of 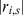 in Equation (1) belongs to the top 10% of 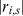 values of all tumor samples, where 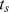 is the expression value of a tumor sample 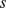 and 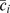 is the mean expression of all control samples for the 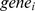. Based on the distributions of 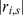 for GBM and OVC data sets, 0.4 was selected for GBM and 0.365 for OVC.
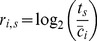
(1)


A manual literature survey provided supportive evidence for the direct relationships in GBM Module 2. Genes in MAPK1-MAPK3, MAPK3-MAPK9, and MAPK1-MAPK9 pairs are involved in various cancer- and GBM-related pathways, including MAPK signaling, ERBB signaling, focal adhesion, and Toll-like receptor signaling. In BRCA2-ING1, both genes play critical roles in cell cycle control [35], [36]; ING1 is a tumor suppressor gene and interacts with TP53, and its under-expression and genetic rearrangement have been observed in several cancers, including GBM [37]; and BRCA2, a tumor suppressor gene, has recently been targeted for sensitizing glioma cells for killing by anti-cancer drugs [38]. In BTBD2-TEP1, TEP1 is a well-known GBM suppressor gene, and the deletion/mutation of this gene has been observed in many cancers, including GBM [39]; polymorphism of BTBD2 is involved in the double-strand break repair pathway that can be useful for GBM survival [40]. In ING1-HMGB1, both genes are located in chromosome 13q, where copy number loss has been reported [41]–[43], suggesting co-occurring deletion of these two genes. In APEX1-HIF1A and HIF1A-TEP1 having the CNA-CNA relationship, APEX1 and HIF1A directly interact with each other *in vitro* [44]; and, in GBM, copy number loss at 14q11.1-q13.1, 14q23.2-q23.3, and 14q32.33, where these genes are located, has been reported by Donovan *et al.* [45]. The relationship between 14q11.1–11.2 and 14q23.1–31.3 are also shown in our findings of CNA-GE relationships (APEX1-BRCA1, BRCA1-HIF1A, and BRCA1-TEP1) within this module. In BTBD2-BARD1, BARD1 was suggested as a mediator of apoptosis since its over-expression induces cell death [46]; and high LOH has been detected in human carcinoma metastases to the brain at chromosome 19p13.3 for BTBD2 [47].

Figure 3B shows enrichment tests using KEGG and BioCarta pathways for the GBM Module 2. To find GBM-related pathways, we also calculated the 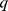-values for the enrichment of GBM-related genes in these pathways, respectively. In Figure 3B, the top 15 of 37 enriched KEGG and the top 15 of 49 enriched BioCarta pathways are shown for the GBM Module 2, along with their corresponding overlapping 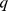-values, sorted by those 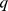-values. GBM Module 2 contains many previously known GBM-related KEGG pathways including Glioma, P53 signaling, MAPK signaling, ERBB signaling, mTOR signaling, and VEGF signaling, and GBM-related BioCarta pathways, including ATM, G2, G1, RB, P53, PTEN, and MET pathways [48]. GBM Module 2 is also enriched with cancer-related 40 KEGG, 48 BioCarta pathways, and 92 GO terms.

We also tested the relevance of GBM Module 2 with cancer using CGC and GBM-related genes, as shown in Figure 3C. GBM Module 2 contained 10 CGC genes of TP53, BRCA1, BRCA2, DAXX, DDX5, MDM2, MDM4, NPM1, TEP1, and WRN, resulting in a 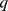-value of 1.05
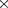
10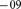, and 2 GBM-related genes of TP53 and TEP1, resulting in a 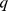-value of 1.02
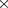
10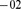.

#### Cancer-related modules identified by the VToD algorithm for ovarian cancer

Among 23 OVC modules, 22 (95.65%), 18 (78.26%), 23 (100%), 15 (65.22%), and 18 (78.26%) modules were significantly enriched (FDR 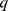-value 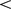 0.05) with at least one KEGG, BioCarta pathways, GO terms, CGC [31], or OVC-related gene sets [34], respectively. Also, 19 (82.61%), 18 (78.26%), and 23 (100%) OVC modules were significantly enriched with cancer-related KEGG, BioCarta, and GO terms, respectively. Table 2 shows the summary of five selected modules ordered by OVC-related gene set enrichment 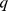-values. All enrichment results for the OVC data set are shown in Tables S8, S9, S10, and S11.

**Table 2:** Summary of functional and cancer gene set enrichments for selected OVC modules (sorted by driver gene set enrichment).

Module ID(Size)	# of enrichedpathways*^a,b,c^*& cancer-relatedpathways*^d,e,f^*	% of gene-gene directrelations*^x,y,z^*	# of CGC & OVCgenes ( 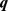 -values)	Enriched cancer genes in modules^‡^
4	41, 54, 102	0.67%	16 (2.60 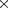 10 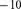 )	AKAP9,AKT1,***APC***,CDH1,
(182)	&	3.05%	11 (8.83 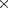 10 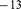 )	***CDKN2A***,***ERBB2***,ERC1,HRAS,
	45, 47, 108	1.05%		MLLT4,***MYC***,NF1,PAFAH1B2,
				PIM1,RAD1,SRGAP3,ZBTB16,
				***AURKA*** 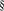 ,***CCNE1*** 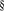 ,***CDKN1A*** 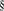 ,
				***DAB2*** 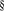 ,***ICAM1*** 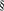 ,***PRKCI*** 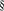 ,***SFN*** 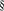
10	48, 92, 82	0.23%	11 (1.95 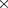 10 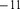 )	AKT1,CARD11,FOXO1,FOXO3,
(51)	&	2.59%	7 (6.48 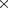 10 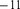 )	JUN,MAP2K4,***MYC***,***PIK3R1***,
	50, 82, 88	9.96%		***PTEN***,PTPN11,TSC1,***VEGFA*** 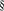 ,
				***CDKN1A*** 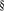 ,***EGF*** 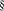 ,***ICAM1*** 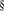
8	37, 59, 41	0.3%	7 (2.08 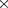 10 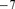 )	PTPN11,AKT1,***ERBB2***,FOXO1,
(37)	&	4.2%	6 (5.23 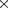 10 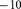 )	HRAS,LIFR,***PIK3R1***,***EGF*** 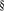 ,
	39, 58, 49	7.36%		***EPHA2*** 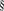 ,***STAT3*** 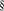 ,***VEGFA*** 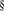
6	41, 23, 128	4.27%	26 (3.78 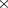 10 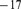 )	AKAP9,***APC***,ATM,BUB1B,
(253)	&	4.27%	9 (5.65 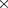 10 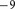 )	CBL,***CDKN2A***,CREBBP,CRTC3,
	38, 23, 122	8.07%		***ERBB2***,ERC1,EZH2,FGFR1OP,
				FOXO3,HSP90AB1,KLF6,MLLT4,
				***MYC***,NF1,NPM1,PAFAH1B2,
				PIM1,***PTEN***,SRGAP3,THRAP3,
				TSC1,ZBTB16,***BCL2L1*** 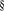 ,***BIRC5*** 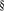
				***DAB2*** 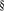 ,***WWOX*** 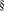
1	47, 95, 95	0.05%	11 (1.78 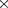 10 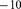 )	***PIK3R1***,ABL1,AKT1,CBL,
(63)	&	1.74%	6 (8.77 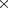 10 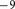 )	CCND3,HRAS,JAK1,MAP2K4,
	47, 86, 92	1.05%		***PTEN***,PTPN11,RB1,***BCL2L1*** 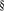 ,
				***EGF*** 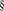 ,***STAT3*** 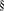 ,***VEGFA*** 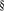

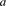
KEGG,

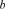
BioCarta,

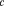
GO Term;

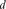
cancer-related subset of KEGG,

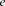
cancer-related subset of BioCarta,

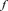
cancer-related subset of GO Term;

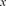
GE-GE,

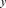
CNA-GE,

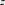
CNA-CNA relationships;

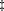
Gene symbols in bold text are OVC-related genes; the remainings are CGC genes.

**Figure 4:**
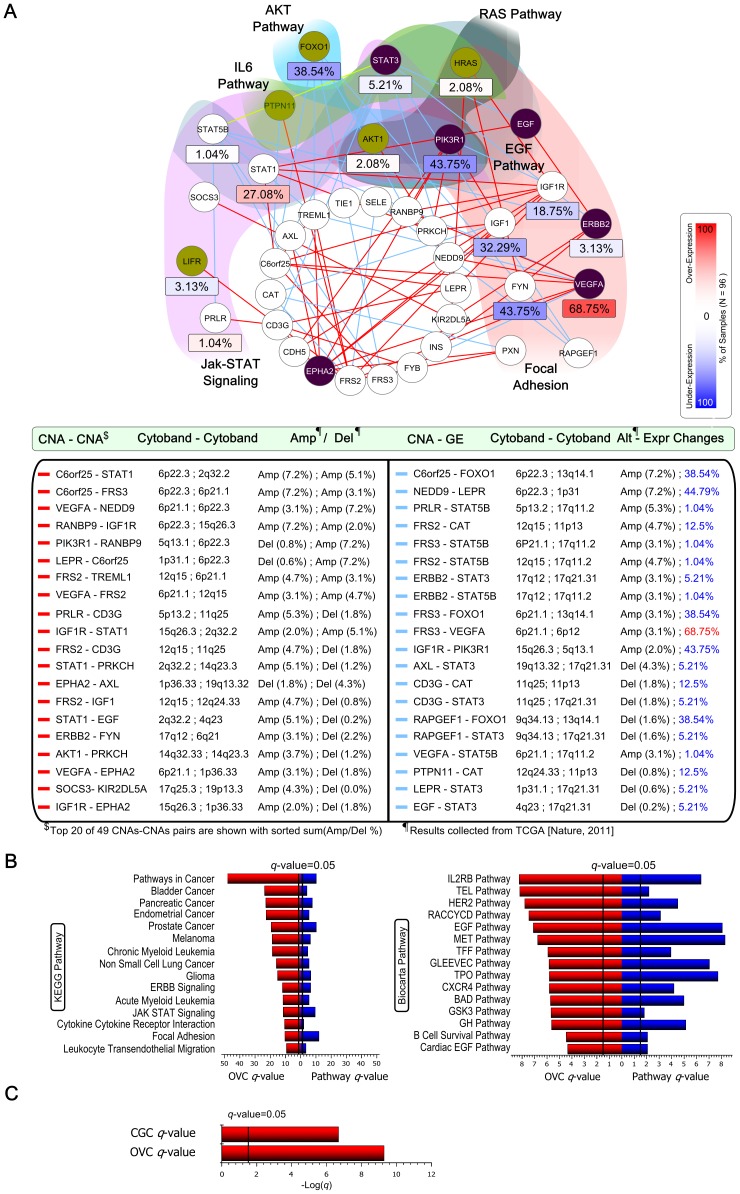
Analysis of OVC Module 8, with a description similar to that of Figure 3. (A) A network view of OVC Module 8 using only direct relationships. CGC genes are colored in olive and OVC-related genes are in purple. (B) Pathway enrichment tests tests were similar to those in Figure 3(B), but here, red bars indicate the overlapping 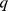-values between the pathway and OVC-related genes. (C) Red bars show the 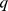-values that overlap with those of the CGC- and OVC-related genes.

We investigated OVC Module 8 in detail, as shown in Figure 4; it contains 629 gene pairs of 37 genes, and among them there were 2 GE-GE, 28 CNA-GE, and 49 CNA-CNA direct relationships. In OVC Module 8, STAT5B-STAT3 gene pair is activated in ovarian cancer [49], interacts with each other [50], and is involved in many pathways including Jak-STAT signaling, RAS signaling, Chemokine signaling, EGF, IL10, PDGF, and TPO pathways. In STAT5B-PRLR, both genes are involved in Jak-STAT signaling, a signal transduction pathway with key control over proliferation, differentiation, and survival of mammary cells [51]. Recently, it has been shown that PRLR and its downstream STAT5B are acetylated by CREB-binding protein (CBP) [52]. In EGF-STAT1 and EGF-STAT3, both gene pairs are involved in pancreatic cancer, EGF pathway, and signal transduction pathway; both STAT1 and STAT3 are activated by the Jak kinase in response to EGF [53]–[55], where JAK2/STAT3 signaling is required for EGF-driven ovarian cancer [55]. In PIK3R1-IGF1R, these genes interact with each other [56] and are involved in many functional pathways, including the IGF1, IGF1R, HDAC, BAD, IGF1MTOR, and focal adhesion pathways. In ERBB2-STAT, these genes are involved in pancreatic cancer and signal transduction pathways; the correlation between the activation of ERBB2 and STAT3 has been observed in many human tumors [57], [58]. In ERBB2-STAT5B, both genes interact with JAK2 [59], [60] and are involved in ERBB signaling and signal transduction pathways. In EGF-ERBB2, these genes directly interact with each other [61] and are involved in many cancers, including pancreatic, endometrial, prostate, bladder and ovarian cancers. They are also involved in ERBB signaling and focal adhesion pathways. In HRAS-FYN, these genes interact with each other *in vitro* [62] and are involved in many pathways, such as focal adhesion, axon guidance, T-cell receptor signaling, and FC epsilon RI signaling, ECM, TCR, and integrin pathways.

The top 15 of 37 enriched KEGG and top 15 of 59 enriched BioCarta pathways are also shown for OVC Module 8 in Figure 4B. It includes known OVC-related KEGG pathways, such as focal adhesion, JAK-STAT signaling, ERBB signaling, cytokine-cytokine receptor interaction, chemokine signaling and VEGF signaling, and OVC-related BioCarta pathways, such as AKT signaling, IL6, RAS, EGF, IGF1, PDGF, VEGF, CXCR4, and HER2 pathways [34]. We also tested the relevance of the OVC Module 8 to cancer. OVC Module 8 was enriched with 39 KEGG, 58 BioCarta pathways, and 49 GO terms, which were cancer-related subsets of the original pathways/terms. Also, as shown in Figure 4C, the OVC Module 8 contained 7 CGC genes (PTPN11, AKT1, ERBB2, FOXO1, HRAS, LIFR, and PIK3R1) with a 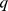-value of 2.08
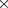
10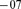 and 6 OVC-related genes (EGF, EPHA2, ERBB2, PIK3R1, STAT3, and VEGFA) with a 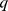-value of 5.23
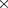
10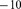. These results suggest that our identified modules from the OVC data set represent cancer-related pathways.

### Comparing VToD with other Methods

Table 3 shows performance comparisons between our proposed VToD algorithm and other clustering methods using GBM and OVC data sets; when compared to these algorithms, a higher fraction of VToD modules were functionally enriched than modules from other algorithms. Although the functional enrichment of DFM-CIN modules is comparable to those of VToD, VToD identified a higher fraction of modules encriched with cancer-related pathways than DFM-CIN. Note that, since algorithms were designed for different data types, they were compared using data types in the original paper. For a hierarchical clustering method, GE, CNAs, and PPI data sets were integrated.

**Table 3:** Comparing VToD to other methods.

Methods	Data setsused	Cancertypes	# ofmodules	# of functionallyenriched modules*^a,b,c^*	# of enriched moduleswith subset ofpathways or terms*^d,e,f^*	# of # distinctpathways orfunctional terms^¶^	# of cancer geneenriched modules^†,‡^
HierarchicalClustering	GE,CNA,PPI	GBM	216	14 (6.48%), 0,13 (6.02%)	4 (1.85%), 0, 4 (1.85%)	51	5 (2.31%), 1 (0.46%)
**VToD**	GE,CNA,PPI	GBM	**22**	**19 (86.36%), 14 (63.63%),** **20 (90.90%)**	**15 (68.18%), 12 (54.55%),** **20 (90.90%)**	**380**	**9 (40.9%), 2 (9.09%)**
		OVC	**23**	**22 (95.65%), 18 (78.26%),** **23 (100%)**	**19 (82.61%), 18 (78.26%),** **23 (100%)**	**508**	**15 (65.22%), 18 (78.26%)**
Cerami et. al.	Mutation,CNA,PPI	GBM	10	1 (10%), 1 (10%),3 (30%)	2 (20%), 1 (10%), 2 (20%)	68	2 (20%), 2 (20%)
MATISSE	GE,PPI	GBM	34	14 (41.18%), 12 (35.29%),12 (35.29%)	4 (11.77%), 1 (2.9%),1 (2.9%)	129	3 (8.82%), 0
		OVC	15	9 (60%), 8 (53.33%),4 (26.67%)	3 (20%), 1 (6.7%), 0	78	7 (46.67%), 2 (13.33%)
**DFM-CIN**	GE,PPI	GBM	**24**	**21 (87.5%), 15 (62.5%),** **24 (100%)**	**5 (20.8%), 4 (1.67%),** **13 (54.17%)**	**429**	**7 (29.17%), 0**
		OVC	**27**	**24 (88.89%),** **17 (62.96%),** **27 (100%)**	**9 (33.33%), 5 (18.52%),** **10 (37.04%)**	**476**	**8 (29.63%), 0**
ClusterONE	PPI Only	–	210	114 (54.29%), 74 (35.24%),119 (56.67%)	100 (47.62%), 72 (34.29%),116 (55.24%)	454	38 (18.09%), 7 (3.33%) 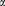 17 (8.09%) 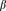

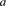
KEGG,

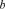
BioCarta,

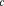
GO Term;

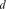
cancer-related subset of KEGG,

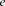
cancer-related subset of BioCarta,

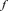
cancer-related subset of GO Term;

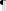
Distinct enriched pathways or terms within all modules were found depending on key terminologies; modules enriched significantly (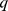-value 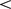 0.05) with 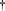
CGC genes and 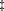
specific cancer-related genes;

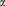
with GBM-related genes and 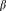
with OVC-related genes.

- Hierarchical clustering: To find modules by the hierarchical clustering algorithm, we converted our gene-gene relationship network 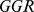 into a distance matrix using the topological overlap metric [63] of the WCGNA tool in the R computational suite. This distance matrix was then used for hierarchical clustering with the average linkage. The dendrogram of the cluster was cut by a dynamic tree-cut [64] algorithm, finally producing 216 modules when the GBM data set was used. We applied functional and cancer gene set enrichment tests with these 216 modules. We found 14, 0, and 13 modules having significant overlaps with KEGG, BioCarta pathways, and GO terms, respectively, and 4, 0, and 4 enriched modules with cancer-related subsets of KEGG, BioCarta, and GO terms, respectively. Also, 5 and 1 modules were enriched with CGC- and GBM-related genes (Table S12). Table 3 shows the comparative performance between hierarchical clustering and VToD algorithms, showing that VToD identified more pathway-enriched modules than the hierarchical clustering algorithm (Table S13). Moreover, Figure S6A shows the box plot of CGC and GBM driver gene enrichment 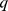-values, indicating higher cancer gene enrichments in VToD compared to hierarchical clustering. Also, the pie charts in Figure S6B show different combinations of three types of direct relationships (CNA-CNA, GE-CNA, GE-GE). Here, VToD produced a larger fraction of modules containing more than one type of direct relationships compared to hierarchical clustering.
- Cerami et. al.: Cerami *et al.* [9] developed an algorithm to integrate DNA copy numbers, somatic mutation, and PPI data sets, and applied it to 84 TCGA GBM data [8]. In their study, altered genes were identified using RAE [12], and a network of genes were constructed based on PPI information using an edge-betweenness algorithm [65], resulting in 10 overlapping modules. When functional and cancer gene set enrichment tests were conducted for these modules, one, one, and three modules were significantly enriched with at least one KEGG, BioCarta pathways, and GO terms, respectively, and 2, 1, and 2 modules were significantly enriched with cancer-related subsets of these three categories of pathways. Also, 2 and 2 modules were significantly enriched with CGC- and GBM-related gene sets, respectively.
- MATISSE: MATISSE [66] integrates gene expression and PPI data sets to find modules, where the apprearance of genes im multiple modules is allowed. We applied MATISSE to both GBM and OVC data sets. Front nodes were filtered based on maximum/minimum fold changes with the 1,000 highest-ranking patterns. Parameters were set as follows: ‘best neighbor seeds’,‘logistics priors as prior regulation’, beta  = 0.95 (default), minimum seed size  = 5 (default), maximum seed size  = 50 (default), and CC = Dot Product (Pearson); and the maximum/minimum sizes of the final clusters were set to ensure that similar numbers of genes were found in the final clusters as those from VToD. Thereupon, we found 34 GBM and 15 OVC modules using the MATISSE method, where 14, 12, 12, 3, and 0 GBM modules and 9, 8, 4, 7, and 2 OVC modules were significantly enriched with KEGG, BioCarta pathways, GO terms, CGC, and cancer type specific gene sets, respectively. Also, enrichment tests of these 34 GBM and 15 OVC modules showed that 4, 1, and 1 GBM modules, and 3, 1, and 0 OVC modules were significantly enriched with cancer-related subsets of KEGG, BioCarta pathways, and GO terms, respectively.
- DFM-CIN: The DFM-CIN [67] method identifies protein complexes and functional modules by combining gene expression and PPI data sets. In their paper, DFM-CIN was compared to and outperformed five other clustering algorithms. Using GBM and OVC data sets, we first applied a TSN-PCD method (sub-method) to find subnetworks using the expression threshold  = 0.7, 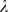  = 1.0 and a minimum complex size of 3 for both GBM and OVC data sets. Next, we applied a DFM-CIN method to detect functional modules based on those subnetworks using similarity threshold  = 0.4, a minimum module size of 3 (for GBM) and of 15 (for OVC), and a maximum module size of 69 (for GBM) and of 252 (for OVC). These parameters were set to produce modules comparable to VToD in terms of the number and size of the modules. Finally, we found 24 GBM and 27 OVC modules. Out of these modules, 21, 15, and 24 GBM modules, and 24, 17, and 27 OVC modules had significant overlap with KEGG, BioCarta pathways, and GO terms, respectively. Also, out of these modules, 5, 4 and 13 GBM modules, and 9, 5 and 10 OVC modules were significantly enriched with cancer-related subsets of these three categories of pathways. Also, 7 GBM modules and 8 OVC modules were enriched with CGC genes, and no modules were enriched with GBM- or OVC-related gene sets.
- ClusterONE: ClusterONE [68] detects clusters in PPI networks with the expansion of overlapping neighborhoods. We applied ClusterONE over our PPI data set with default parameter settings and found 210 clusters of genes. The similar enrichment tests yielded 114, 74, 119, 38, 7, and 17 clusters with significant overlaps with KEGG, BioCarta pathways, GO terms, CGC, GBM- and OVC-related genes, respectively. Also, 100, 72, and 116 clusters were significantly enriched with cancer-related subsets of KEGG, BioCarta pathways, and GO terms, respectively.

DFM-CIN [67] was comparable to VToD and outperformed other competing methods in functional enrichment tests for both GBM and OVC data sets. However, VToD outperformed all other methods in terms of cancer gene set enrichment tests and cancer-related pathway enrichment tests for both GBM and OVC data sets, indicating that identified modules were more likely to be related to the cancer. The numbers of distinct pathways or functional terms enriched for VToD modules were comparable to DFM-CIN and greater than those of other methods, showing the convincing performance of our algorithm. All distinct enriched pathways or terms found by the above methods, including VToD for both GBM and OVC data sets, are shown in Tables S13 and S14, respectively.

## Discussion and Conclusions

We proposed the voting-based module construction approach by integrating three direct relationships (GE-GE, CNA-GE, and CNA-CNA), along with indirect relationships and PPI information. We have shown that our relationship network by integrating GE-GE, CNA-GE, and CNA-CNA types can be useful for giving explainable relationships between genes in identified modules since most of the modules contained different types of relationships; by observing CGC enrichment result, all 9 GBM modules and 14 of 15 OVC modules constructed by the VToD algorithm contain at least two types of direct relationships, implying that GE changes and CNAs occur simultaneously in cancer modules. This conclusion was further confirmed when we found that the numbers of different types of direct relationships in modules had strong a positive correlation with CGC enrichment 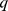-values (0.64 for GBM and 0.49 for OVC) and module sizes (0.67 for GBM and 0.52 for OVC).

In this study, we combined both data-driven and topological properties throughout the whole algorithm, from constructing pre-modules to merging pre-modules. However, our approach has limitations in combining these two properties. When we combined the data-driven and topological properties to calculate vote-values among gene pairs, we integrated them using the same weights (see Equation (4) in the Methods section), although the distribution and the contribution of each property might be different. For further investigation, we drew distributions of topological values and data-driven values of gene pairs contained in the pre-modules of GBM, as shown in Figures S7A and S7C, respectively. The distributions of these two values were different; a Kolmogorov-Smirnov (K-S) test under the null hypothesis that two distributions are identical gives a 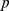-value of 2.2e-16. Similar results were observed in OVC, as shown in Figures S7B and S7D. However, when we drew scatter plots of data-driven property values and topological property values of gene pairs included in the GBM and OVC pre-modules (Figures S7E and S7F), one property was not dominated by the other property. In many gene pairs, one of two properties had a relatively larger value while the other had relatively smaller value, showing negative correlations between them (−0.550 for GBM and −0.259 for OVC). This observation showed that both properties were significantly contributing to constructing pre-modules.

When we combined the data-driven and topological properties to merge two pre-modules, we also integrated them using the same weights (see Equation (5) in the Methods section). Distributions of topological values and data-driven values of all pairs of pre-modules are different (Figure S8). One interesting observation is that most pairs of pre-modules have value one for the topological property for both GBM and OVC data sets, as shown in Figures S8A and S8C, respectively. Consequently, most merged pre-modules have value one for the topological property (Figures S9A and S9B for GBM and OVC, respectively), and values of the data-driven property in those modules is also high. According to these observations, different distributions of these two properties might not significantly reduce the performance of the proposed algorithm.

Our method of combining two different distributions can be further improved; for example, (i) a distribution is transformed into the standard normal distribution, and then (ii) the optimal contribution weights of two distributions is searched. However, a new weight parameter adds an additional complexity to the model. The advantages of the current approach are parameter-less and intuitively simple, and the comparative assessments showed that our methods outperformed other methods in detecting cancer-related modules.

Our primary goal in this study was to establish the relationships among genes in cancer pathways using an integrated approach. We hope that our research will help explain complex relationship between genes in cancer development. Although we validated the identified modules by using functional and cancer gene set enrichment tests in the current study, more experiments, such as survival analysis and the classification of normal/tumor patients, will be part of our future work.

## Materials and Methods

### Data Sets

GE and CNA (level 3) data from 266 paired GBM and 96 paired OVC tumor samples were downloaded from the TCGA data portal (as of 24 July 2011 and 25 June 2012, respectively). GE data were measured using the Affymetrix Human Genome U133 array platform. For both cancer data sets, expression values for 12,044 genes were organized as GE data matrices, labeling rows with gene symbols and columns with sample identifiers (Figure 1A). DNA copy numbers were measured using Agilent's Human Genome CGH microarray 244A platform. To obtain gene-level CNA values, segmented regions in the level 3 data (for both GBM and OVC data sets) except sex chromosomes (Chr X and Y) were mapped to gene symbols (Table S1); gene symbols were mapped to segmented regions using refGene.txt (version hg18), downloaded from the UCSC genome browser, and ambiguous annotations (genes with multiple annotations) of 28 genes were manually resolved by using either NCBI, the Encode Gencode manual, a BLAT similarity/score (26 genes), or refGene.txt of version hg19 (2 genes). Then, CNA data matrices for both GBM and OVC data sets were organized with the same tumor samples (columns) as in corresponding GE data matrices, and 22,082 and 22,086 genes (rows), respectively. Missing values in CNA matrices were imputed with the mean across all samples. PPI information was collected from [9], including i) manually curated interactions in HPRD [69], ii) signaling pathways from Reactome and NCI/Nature pathway interactions, and iii) the MSKCC Cancer Cell Map. We further converted this PPI information into an undirected graph with 44,959 genes as nodes and 96,347 pairwise interactions as edges.

### Constructing a Gene-gene Relationship Network

We define a gene-gene relationship network as *GGR*: =  (*S*, *R*). Here, *S* is a set of seed genes and *S*: =  (*DE* 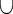 *SA*), where *DE* and *SA* are sets of differentially expressed and significantly copy number altered genes, respectively. *R* is the set of pairwise gene-gene relationships among these seed genes (Figure 1B).

#### Seed genes

To find the *DE* genes in *S*, the two-tailed pooled 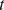-test was used. For the 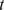-test, 10 and 8 unmatched normal samples downloaded from the GBM and OVC pages of the TCGA data portal were used as control data sets, respectively. 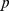-values were corrected by the Bonferroni correction method and genes with corrected 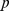-values below a threshold were selected. To find *SA* genes in GBM, we collected the focal aberrant regions identified by the GISTIC and RAE algorithms in [8], where a subset of samples of our study were used (Table S3 and S4 of the original article, respectively). In these two algorithms, focal aberrant regions were detected to distinguish relatively short aberrant regions containing cancer-related genes from random broad aberrant regions. Similarly, we collected altered regions found by GISTIC in [14] (Supplementary Table S5.2 of the original article) for OVC. Some genes in *S* had only GE data, some had only CNA data, and others had both GE and CNA data (shown in open rectangles, open circles, and filled circles in Figure 1B, respectively).

#### Relationships among gene pairs

Pairwise relationships between genes are measured by using an absolute value of Pearson correlation coefficient (PCC). For any gene pair (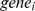, 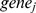), absolute PCC values from GE-GE, CNA-GE, and CNA-CNA data are calculated depending on data availability. Since any type of relationship between two genes might affect cancer development, all three absolute PCCs are considered, and the maximum of them was chosen as a potential relationship value and defined as a 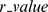 (Figure 1B). Note that, since proximal genes on the chromosome are frequently amplified or deleted together, gene pairs were not considered the CNA-CNA relationship if they were in the same focal aberrant regions or within the same cytoband.

A gene pair (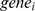, 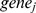) is included in the gene-gene relationship *R* with the 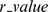 as a weight if the 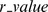 is larger than a threshold, and the relationship is called a direct relationship. The threshold is empirically chosen based on the distribution of all PCC values (see the Parameter selection section below). For each gene pair (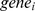, 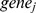) 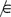 *R*, indirect relationships are found by searching a statistically significant simple path between two genes. Here, the significant path between two genes is defined as a list of other genes that gives the statistically significant geometric mean of 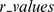 from 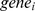 to 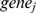 throughout genes in the list compared to the geometric mean of the path in a random PPI network. The random PPI network is generated such that interactions between genes are randomly assigned, while the topology of the network and expression values of genes are conserved with those in the observed PPI network. The null hypothesis for the statistical significance test is that the geometric mean of 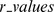 of a simple path in the randomly generated PPI network is greater than or equal to that of the observed path. However, since it takes an exponential time to consider every possible pathes between two genes, a heuristic search is developed; paths between 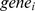 and 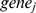 are searched if two genes are connected in the PPI graph. A breadth first search algorithm is used to search all simple paths between two genes (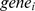, 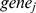) 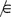 *R*. Also, only the paths in which all genes have either GE, CNA, or both types of data are considered. Since searching might yield multiple paths, we chose the path 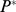 with the maximum average PPI connectivity, since genes having larger interactions with other genes are more likely to be related to cancer (see File S1). 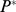 is measured by Equation (2),
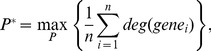
(2)where 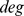
(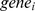) is the degree of connectivity in the PPI graph for 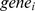 and 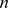 is the number of genes along the path 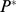. Before calculating the average connectivity for the genes along the path, the connectivity of each gene 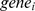 is normalized with the global maximum PPI connectivity (Equation (3)) to make the value in the range of [0, 1].



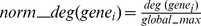
(3)Then, the statistical significance of the path P* between 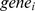 and 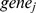 is assessed based on the randomly generated PPI network. In the null hypothesis mentioned above, the observed value is the geometric mean of all pairwise 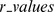 along the path P*. If 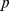-value of the path P* obtained by comparing to 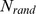 random PPI networks is below a threshold, the gene pair (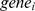, 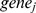) is included in the gene-gene relationship network *R* as the *indirect* relationship.

### Module Detection: VToD

The proposed algorithm is described in Table 4, and source codes of the VToD algorithm is provided in http://www.gcancer.org/VToD/VToD.html.

**Table 4:** The VToD algorithm.

	VToD (*GGR*,*PPI*)
	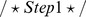
1	**for** each gene 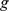 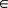 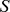
2	**for** each gene 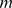 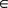 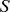 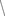 { 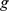 }
3	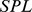 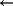 the shortest path length between gene 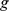 and 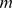
4	**if**( 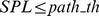 ) then
5	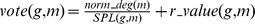
6	**else**
7	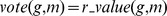
8	**end if**
9	**end for**
10	**end for**
	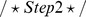
11	**for** each gene 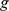 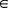 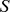
12	**for** each gene 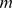 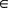 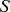
13	Calculate a local rank and a global rank for 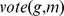
14	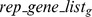 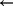 Select gene 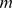 whose local rank and global rank of 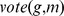 are within 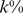 and 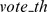 %, respectively.
15	**end for**
16	**end for**
	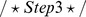
17	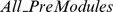 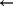 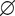
18	**for** each gene 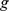 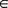 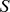
19	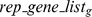 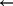 List of representative genes for ‘ 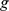 ’
20	**for** each gene 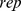 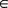 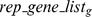
21	**if**( 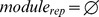 ) then
22	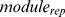 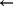 Make a new pre-module
23	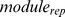 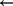 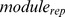 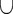 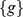
24	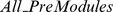 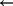 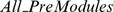 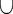 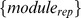
25	**else**
26	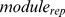 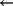 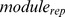 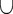 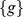
27	**end if**
28	**end for**
29	**end for**
30	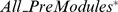 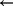 Remove Redundancy
	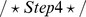
31	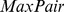 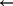 FindMaxPair( 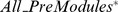 )
32	**while**( 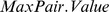 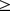 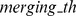 )
33	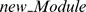 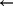 Merge two modules in 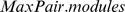
34	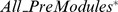 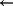 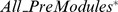 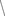 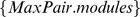
35	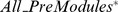 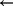 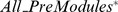 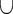 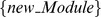
36	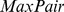 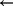 FindMaxPair( 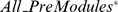 )
37	**end while**

#### Step 1: Calculate the association between genes

In VToD, pairwise votes are first calculated for every pair of genes 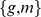 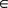 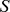, as shown in Equation (4), where 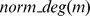 is the normalized PPI connectivity of 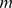 using Equation (3), 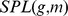 is the shortest path length between two genes in the PPI graph, and 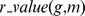 is the relationship value used from our constructed network *GGR*.
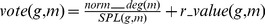
(4)


Equation (4) calculates the score when a gene 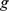 chooses a gene 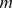 as a representative gene; the score increases if (i) a gene pair 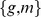 has a high relationship 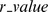, denoting data-driven property, or (ii) the gene 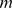 with a high topological value is closely interacting with the gene 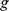 in the PPI network. Hub genes in the PPI network have more chances to be selected as representative genes due to 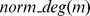, but are controlled by the length of the shortest path between 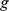 and 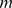 to produce functional modules related to the gene 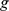. We apply constraints to the shortest path length value 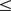 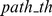 to increase the compactness of a pre-module, and to reduce the time-complexity for searching the shortest path. If the shortest path length between the gene 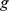 and the gene 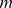 is larger than 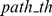, the topological information is not considered and the vote-value is defined using only the data-driven value between them. Note that 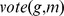 has values between 0 and 2 since both terms have values between 0 and 1.

#### Step 2: Select representative genes of each gene

After the vote calculation, a gene 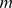 is selected as a representative gene of a given gene 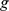 based on a local rank and a global rank. In calculating the local rank of the gene 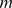 for the gene 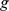, all genes in 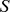 are ranked by descending order of vote-values to the gene 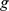. Then, the cumulated vote-value from the largest vote-value to the vote-value of the gene 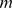 is calculated. If the cumulated vote-value is within the top 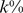 of all cumulative vote-values, the gene 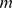 is considered a candidate representative gene of the gene 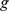. For the global rank, if the 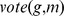 value is located within the top 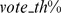 of vote-values between all gene pairs in 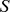, the gene 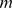 is selected as the representative gene of the given gene 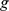. This approach allows multiple representative genes for the gene 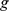, and one gene can be selected as a representative gene for multiple genes.

#### Step 3: Forming pre-modules

Each representative gene 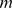 (from Step 2) starts forming a pre-module including only itself. Then, each module is enlarged by aggregating all genes that selected the gene 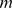 as the representative gene. A redundant pre-module is removed when it is a subset of other pre-modules. Smaller pre-modules are also removed if they contain either only two genes (including the representative gene) or all the genes except the representative gene in the pre-module are located in the same focal region of chromosomes.

#### Step 4: Merging pre-modules

In this step, two pre-modules are merged if pairwise members of the two pre-modules are highly related in the gene-gene relationship network and are closely connected in the PPI network. A pairwise merging value MV(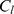, 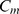) between any two pre-modules 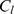 and 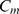 is calculated by Equation (5). Let the sizes of 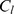 and 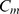 be 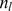 and 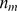, respectively, and let 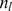 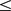 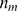. In the equation, the topological property between two pre-modules (or modules) is given as the ratio of genes in 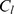 having at least one protein interaction partner in 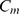 (inter-connectivity: IC(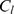, 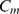)). Data-driven properties are calculated as the average of gene-gene relationship values between two pre-modules (or modules).
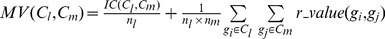
(5)


At every merging step, two modules having the maximum pairwise merging value, denoted as 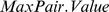 in Step 4 of Table 4, are merged and replaced by the newly merged module in the module set. Such merging processes continue until the 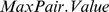 is below the threshold 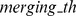 that is decided by comparing the merging values generated by randomized modules (see the next section for generating a randomized module).

### Statistical Significance of the Identified Modules

The statistical significance of the identified modules is validated by comparing them to randomized modules. To generate randomized modules, 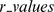 of gene pairs in the gene-gene relationship network 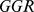 are shuffled, while the PPI network remains unchanged, so that the topological property is disconnected from the data-driven property; then, using this shuffled relationship network 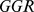, the whole VToD algorithm from Step 1 to Step 4 runs until a single pre-module is left. This generation of randomized modules were repeated 100 times. Concurrently, the observed pre-modules were merged until a single pre-module is left. At each merging step, a pre-module pair that yields the maximum merging value is selected, and these values for both observed and random cases are plotted in Figures S3A and S3C, for GBM and OVC, respectively. At each merging step, the observed value was significantly greater than the average of the maximum merging values in random cases, confirming that the modules identified by the VToD algorithm are statistically significant.

Next, to find the merging threshold 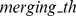, the stopping criteria of merging pre-modules, for each merging step, we compared the maximum merging value of the observed case with the maximum merging values of the first merging step in all 100 random cases and measured the empirical 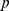-value (Figures S3B and 3D for GBM and OVC data sets, respectively).

### Functional and Cancer Gene Set Enrichment

We tested whether constructed modules from both GBM and OVC data sets were enriched with known signaling pathways or biological functions. We used 186 KEGG pathways, 217 BioCarta pathways, and 751 biological processes in GO downloaded from the Molecular Signature Database (MsigDB) at the Broad Institute (http://www.broad.mit.edu/gsea/msigdb/msigdb_index.html). We excluded GO terms with sizes 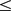 5 and 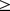 250 to omit too-specific or too-general terms. Next, we selected cancer-related subsets of pathways/terms from all pathways/terms. To find such pathways/terms, we measured statistically significant enrichment of cancer genes from CGC [31] in pathways/terms by applying a hypergeometric test and by correcting the 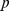-values using the FDR multiple comparison correction, giving 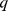-values. By applying 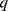-values 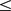 0.05, 83, 139, and 338 cancer-related pathways/terms were selected from KEGG, BioCarta pathways, and GO biological process terms, respectively; they are listed in Table S15. Also, cancer genes from CGC [31], GBM-related genes [33], and OVC-related genes [34] were used to measure the cancer gene enrichment of the identified modules.

For the enrichment analysis, a hypergeometric test was applied to each module using the above all pathways/terms, cancer-related pathways/terms, and cancer gene sets, giving 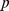-values, and 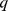-values were obtained by the FDR multiple comparison correction. The 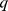-values 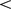 0.05 was used for an enrichment threshold. Note that 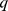-values depend on the number of comparisons and 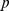-values of comparisons in the enrichment test. Therefore, it may happen that although a module is enriched for a pathway when the multiple comparison correction was performed using the cancer-related subset of pathways, the module is not enriched for the same pathway when the correction was done using all pathways, and vice versa.

### Parameter Selection

Our algorithm has following parameters: thresholds for selecting differentially expressed genes, thresholds for 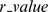, 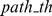 for searching indirect relationships, 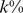 and 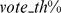 for selecting representative genes, and 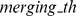 for merging pre-modules. We used 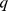-value 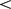 0.05 for selecting differentially expressed genes (Bonferroni corrected) and 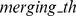. 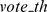 were tested for three different values, as shown in the Results section. However, 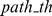, thresholds for 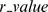, and 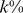 were empirically chosen since these parameters affect the intermediate steps and are not critical for final modules. Here, we explain these parameters in detail.

From the distribution of three different direct relationships (GE-GE, CNA-GE, and CNA-CNA), the top 10% of all corresponding PCC values were selected as thresholds: 0.38, 0.165, and 0.435 for GE-GE, CNA-GE, and CNA-CNA relationships, respectively, for GBM (Figure S1A). By applying corresponding thresholds to the 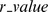
s between any pair of genes, 2,617,259 direct relationships were included in the gene-gene relationship 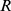, which was 22.53% of 11,618,610 total gene pairs consisting of 4,821 seed genes in 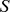.

To search indirect relationships for any pair (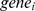, 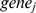) 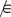 *R*, the most relevant path 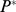 was chosen after exploring all simple paths in the PPI graph with 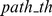  = 2. The geometric mean of pairwise 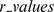 along 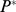 was calculated and the statistical significance was measured over 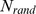 ( = 50) randomly generated PPI networks. A gene pair (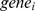, 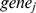) having a 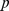-value 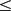 0.05 was considered an indirect relationship and added to the gene-gene relationship 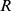. The 42,532 total pairs updated by indirect relationships were 0.47% of all 9,001,351 ( = 11,618,610–2,617,259) gene pairs.

Using an experimental setup similar to the one above, we selected 0.295, 0.19, and 0.19 as thresholds for GE-GE, CNA-GE, and CNA-CNA direct relationships, respectively, for OVC (Figure S1B). Applying these thresholds to 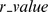
s, we defined 5,681,333 (25.71% of all pairs) direct relationships in 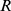, followed by updating 52,969 pairs (0.32% of all remaining pairs in 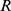) as indirect relationships.

To select representative genes for each gene, we needed to decide two thresholds, 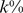 and 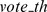
%; the 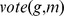 value is located (i) within the top 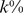 of local vote-values and (ii) within the top 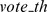
% of global vote-values. We used 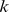 = 1 and tested three values for 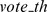 of the top 1%, 0.25%, and 0.1%, as mentioned in the Results section. The distributions of all votes for the GBM and OVC data sets are shown in Figure S2.

## Supporting Information

Figure S1
**Distributions of all enumerated pairwise direct relationships among the genes in 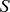.** (A) is for GBM data set and (B) is for OVC data set. X-axis indicates the absolute Pearson correlation coefficient (PCC) for GE-GE, CNA-GE and CNA-CNA relationships. For each distributions, y-axis indicates the proportion of gene-pairs among the total number of pair-wise relationships (GE-GE, CNA-GE, and CNA-CNA) having corresponding PCC values. Here, we show the selection of individual thresholds in the distribution using the arrows. For both data sets, several peaks were observed, but we did not find any particular reason for these peaks. Since we used a binning approach to draw distributions, the observed peaks depend on the bin size. For our convenience, we used the bin size of 0.01.(EPS)

Figure S2 **Distribution of pairwise voting values among genes.** (A) is for the GBM data set and (B) for OVC data set. X-axis and y-axis show the vote-values and their corresponding frequencies among all gene pairs.
(EPS)

Figure S3
**Statistical validation of the identified modules and selection of 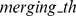.** (A) and (C) show comparison between merging values between the observed case and 100 random cases, for GBM and OVC data sets, respectively. (B) and (D) show 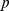-values for merging values at each merging step, for GBM and OVC data sets, respectively.(EPS)

Figure S4 **Module overlaps in terms of common genes.** In (A) and (C), the ratios of the number of common (overlapping) genes among the number of genes in the module are shown in x-axis, for GBM and OVC data sets, respectively. Frequencies of modules with the corresponding overlapping ratio in x-axis is shown in y-axis. In (B) and (D), the average ratios of overlapping genes in KEGG, BioCarta, and VToD are shown for GBM and OVC data sets, respectively.
(EPS)

Figure S5 **Representation of fractions of gene pairs having direct relationships in modules.** The x-axis shows the module ID and the y-axis shows the fractions of gene pairs having each type of direct relationships out of all possible gene pairs for 22 GBM modules (A) and 23 OVC modules (B). For a particular module, there are three vertical bars; a blue vertical bar shows the fraction for the GE-GE relationship, a red bar for the CNA-GE relationship, and a green bar for the CNA-CNA relationship. For a gene pair, all three types of direct relationships can be above their corresponding thresholds. Therefore, the fraction of each individual vertical bar in a module is at most 1. For example, GBM module 2 has 48 genes, indicating 1,128 gene pairs. Among 1,128 gene pairs, there are 300 (300/1,128 = 0.2659) gene-pairs with the GE-GE relationship, 9 (9/1,128 = 0.0079) gene pairs with the CNA-GE relationship, and 8 (8/1,128 = 0.007092) gene pairs with the CNA-CNA relationship.
(EPS)

Figure S6 **Comparison between hierarchical clustering and the VToD algorithm.** (A) Box charts of CGC and GBM driver gene set enrichments for both the hierarchical clustering and the VToD algorithm. (B) Percentages of modules in the hierarchical clustering and the VToD algorithm that contain different combinations of all three types of direct relationships.
(EPS)

Figure S7 **Topological and data-driven properties used in the vote calculation.** (A) and (B) show the distributions of topological property values of gene-pairs included in pre-modules, for GBM and OVC, respectively. (C) and (D) show the distributions of data-driven property values of gene-pairs included in pre-modules, for GBM and OVC, respectively. In the inset panel, the distribution of each property (both topological and data-driven) values of all gene-pairs is shown. (E) and (F) are scatter plots of data-driven property values versus topological property values of gene-pairs included in the pre-modules, for GBM and OVC, respectively.
(EPS)

Figure S8 **Distributions of topological and data-driven properties in merging pre-modules.** (A) and (C) are distributions of topological property values of all pairs of pre-modules, and (B) and (D) are distributions of data-driven property values of all pairs of pre-modules, for GBM and OVC, respectively.
(EPS)

Figure S9 **Topological and data-driven property values while merging pre-modules.** (A) and (B) show contributions of both properties to calculate merging-values while merging pre-modules for GBM and OVC data sets, respectively. Topological properties are colored in black and data-driven properties are in blue.
(EPS)

Table S1 **CNA genes and locations.**
(XLSX)

Table S2 **List of identified modules for GBM.**
(XLSX)

Table S3 **List of identified modules for OVC.**
(XLSX)

Table S4 **Summary of enrichment test results for GBM modules (VToD algorithm).**
(XLSX)

Table S5 **Enrichment test results of KEGG pathways for GBM modules (VToD algorithm).**
(XLSX)

Table S6 **Enrichment test results of BioCarta pathways for GBM modules (VToD algorithm).**
(XLSX)

Table S7 **Enrichment test results of GO biological processes for GBM modules (VToD algorithm).**
(XLSX)

Table S8 **Summary of enrichment test results for OVC modules (VToD algorithm).**
(XLSX)

Table S9 **Enrichment test results of KEGG pathways for OVC modules (VToD algorithm).**
(XLSX)

Table S10 **Enrichment test results of BioCarta pathways for OVC modules (VToD algorithm).**
(XLSX)

Table S11 **Enrichment test results of GO biological processes for OVC modules (VToD algorithm).**
(XLSX)

Table S12 **Summary of enrichment test results for GBM modules (Hierarchical clustering).**
(XLSX)

Table S13 **Comparison of distinct enriched pathways between other methods and VToD for GBM data set.**
(XLSX)

Table S14 **Comparison of distinct enriched pathways between other methods and VToD for OVC data set.**
(XLSX)

Table S15 **Cancer-related KEGG pathways, BioCarta pathways, and GO biological processes.**
(XLSX)

File S1 **Descriptions about finding indirect relationships, and topological and data-driven properties in merging pre-modules.**
(PDF)

## References

[1] Hahn WC, Weinberg RA (2002) Modelling the molecular circuitry of cancer. Nat Rev Cancer 2: 331–341. 12044009 10.1038/nrc795

[2] Vogelstein B, Kinzler KW (2004) Cancer genes and the pathways they control. Nat Med 10: 789–799. 15286780 10.1038/nm1087

[3] Davies H, Bignell GR, Cox C, Stephens P, Edkins S, et al (2002) Mutations of the BRAF gene in human cancer. Nature 417: 949–954. 12068308 10.1038/nature00766

[4] Wan PTC, Garnett MJ, Roe SM, Lee S, Niculescu-Duvaz D, et al (2004) Mechanism of Activation of the RAF-ERK Signaling Pathway by Oncogenic Mutations of B-RAF. Cell 116: 855–867. 15035987 10.1016/s0092-8674(04)00215-6

[5] Santarosa M, Ashworth A (2004) Haploinsufficiency for tumour suppressor genes: when you don't need to go all the way. Biochimica et Biophysica Acta (BBA) - Reviews on Cancer 1654: 105–122. 15172699 10.1016/j.bbcan.2004.01.001

[6] Chuang HY, Lee E, Liu YT, Lee D, Ideker T (2007) Network-based classification of breast cancer metastasis. Mol Syst Biol 3: 140. 17940530 10.1038/msb4100180 PMC2063581

[7] Vaske CJ, Benz SC, Sanborn JZ, Earl D, Szeto C, et al (2010) Inference of patient-specific pathway activities from multi-dimensional cancer genomics data using PARADIGM. Bioinformatics 26: i237–i245. 20529912 10.1093/bioinformatics/btq182 PMC2881367

[8] TCGA (2008) Comprehensive genomic characterization defines human glioblastoma genes and core pathways. Nature 455: 1061–1068. 18772890 10.1038/nature07385 PMC2671642

[9] Cerami E, Demir E, Schultz N, Taylor BS, Sander C (2010) Automated Network Analysis Identifies Core Pathways in Glioblastoma. PLoS ONE 5: e8918. 20169195 10.1371/journal.pone.0008918 PMC2820542

[10] Feuk L, Carson AR, Scherer SW (2006) Structural variation in the human genome. Nat Rev Genet 7: 85–97. 16418744 10.1038/nrg1767

[11] Beroukhim R, Getz G, Nghiemphu L, Barretina J, Hsueh T, et al (2007) Assessing the significance of chromosomal aberrations in cancer: Methodology and application to glioma. Proceedings of the National Academy of Sciences 104: 20007–20012. 10.1073/pnas.0710052104 PMC2148413 18077431

[12] Taylor BS, Barretina J, Socci ND, DeCarolis P, Ladanyi M, et al (2008) Functional Copy-Number Alterations in Cancer. PLoS ONE 3: e3179. 18784837 10.1371/journal.pone.0003179 PMC2527508

[13] Hur Y, Lee H (2011) Wavelet-based identification of DNA focal genomic aberrations from single nucleotide polymorphism arrays. BMC Bioinformatics 12: 146. 21569311 10.1186/1471-2105-12-146 PMC3114745

[14] TCGA (2011) Integrated genomic analyses of ovarian carcinoma. Nature 474: 609–615. 21720365 10.1038/nature10166 PMC3163504

[15] Jornsten R, Abenius T, Kling T, Schmidt L, Johansson E, et al.. (2011) Network modeling of the transcriptional effects of copy number aberrations in glioblastoma. Mol Syst Biol 7. 10.1038/msb.2011.17 PMC3101951 21525872

[16] Akavia UD, Litvin O, Kim J, Sanchez-Garcia F, Kotliar D, et al (2010) An Integrated Approach to Uncover Drivers of Cancer. Cell 143: 1005–1017. 21129771 10.1016/j.cell.2010.11.013 PMC3013278

[17] Witten DM, Tibshirani R, Hastie T (2009) A penalized matrix decomposition, with applications to sparse principal components and canonical correlation analysis. Biostatistics 10: 515–534. 19377034 10.1093/biostatistics/kxp008 PMC2697346

[18] Horvath S, Zhang B, Carlson M, Lu KV, Zhu S, et al (2006) Analysis of oncogenic signaling networks in glioblastoma identifies ASPM as a molecular target. Proceedings of the National Academy of Sciences 103: 17402–17407. 10.1073/pnas.0608396103 PMC1635024 17090670

[19] Choi JK, Yu U, Yoo OJ, Kim S (2005) Differential coexpression analysis using microarray data and its application to human cancer. Bioinformatics 21: 4348–4355. 16234317 10.1093/bioinformatics/bti722

[20] Mo W, Fu X, Han X, Yang G, Zhang J, et al (2009) A stochastic model for identifying differential gene pair co-expression patterns in prostate cancer progression. BMC Genomics 10: 340. 19640296 10.1186/1471-2164-10-340 PMC2737000

[21] Gorringe KL, George J, Anglesio MS, Ramakrishna M, Etemadmoghadam D, et al (2010) Copy Number Analysis Identifies Novel Interactions Between Genomic Loci in Ovarian Cancer. PLoS ONE 5: e11408. 20844748 10.1371/journal.pone.0011408 PMC2937017

[22] Klijn C, Bot J, Adams DJ, Reinders M, Wessels L, et al (2010) Identification of Networks of Co-Occurring, Tumor-Related DNA Copy Number Changes Using a Genome-Wide Scoring Approach. PLoS Comput Biol 6: e1000631. 20052266 10.1371/journal.pcbi.1000631 PMC2791203

[23] Cui Q (2010) A Network of Cancer Genes with Co-Occurring and Anti-Co-Occurring Mutations. PLoS ONE 5: e13180. 20957180 10.1371/journal.pone.0013180 PMC2949398

[24] Masica DL, Karchin R (2011) Correlation of Somatic Mutation and Expression Identifies Genes Important in Human Glioblastoma Progression and Survival. Cancer Research 71: 4550–4561. 21555372 10.1158/0008-5472.CAN-11-0180 PMC3129415

[25] Lee H, Kong SW, Park PJ (2008) Integrative analysis reveals the direct and indirect interactions between DNA copy number aberrations and gene expression changes. Bioinformatics 24: 889–896. 18263644 10.1093/bioinformatics/btn034 PMC2600603

[26] Solvang H, Lingjaerde O, Frigessi A, Borresen-Dale AL, Kristensen V (2011) Linear and non-linear dependencies between copy number aberrations and mRNA expression reveal distinct molecular pathways in breast cancer. BMC Bioinformatics 12: 197. 21609452 10.1186/1471-2105-12-197 PMC3128865

[27] Xu C, Liu Y, Wang P, Fan W, Rue T, et al (2010) Integrative analysis of DNA copy number and gene expression in metastatic oral squamous cell carcinoma identifies genes associated with poor survival. Molecular Cancer 9: 143. 20537188 10.1186/1476-4598-9-143 PMC2893102

[28] Kanehisa M, Goto S, Sato Y, Furumichi M, Tanabe M (2012) KEGG for integration and interpretation of large-scale molecular data sets. Nucleic Acids Res 40: D109–14. 22080510 10.1093/nar/gkr988 PMC3245020

[29] BioCarta. Available: http://www.biocarta.com/. Accessed 2011 Oct 19.

[30] Ashburner M, Ball C, Blake J, Botstein D, Butler H, et al (2000) Gene ontology: tool for the unification of biology. The Gene Ontology Consortium. Nat Genet 25: 25–9. 10802651 10.1038/75556 PMC3037419

[31] Futreal PA, Coin L, Marshall M, Down T, Hubbard T, et al (2004) A census of human cancer genes. Nat Rev Cancer 4: 177–183. 14993899 10.1038/nrc1299 PMC2665285

[32] Hwang W, Cho YR, Zhang A, Ramanathan M (2006) A novel functional module detection algorithm for protein-protein interaction networks. Algorithms for Molecular Biology 1: 24. 17147822 10.1186/1748-7188-1-24 PMC1764415

[33] Parsons DW, Jones S, Zhang X, Lin JCH, Leary RJ, et al (2008) An Integrated Genomic Analysis of Human Glioblastoma Multiforme. Science 321: 1807–1812. 18772396 10.1126/science.1164382 PMC2820389

[34] Bast RC, Hennessy B, Mills GB (2009) The biology of ovarian cancer: new opportunities for translation. Nat Rev Cancer 9: 415–428. 19461667 10.1038/nrc2644 PMC2814299

[35] Sarela A, Farmery S, Markham A, Guillou P (1999) The candidate tumour suppressor gene, ing1, is retained in colorectal carcinomas. European Journal of Cancer 35: 1264–1267. 10615239 10.1016/s0959-8049(99)00104-5

[36] Nakanishi A, Han X, Saito H, Taguchi K, Ohta Y, et al (2007) Interference with brca2, which localizes to the centrosome during s and early m phase, leads to abnormal nuclear division. Biochemical and Biophysical Research Communications 355: 34–40. 17286961 10.1016/j.bbrc.2007.01.100

[37] Tallen G, Farhangi S, Tamannai M, Holtkamp N, Mangoldt D, et al (2009) The inhibitor of growth 1 (ING1) proteins suppress angiogenesis and differentially regulate angiopoietin expression in glioblastoma cells. Oncol Res 18: 95–105. 20066899 10.3727/096504009789954645

[38] Quiros S, Roos WP, Kaina B (2011) Rad51 and brca2 - new molecular targets for sensitizing glioma cells to alkylating anticancer drugs. PLoS ONE 6: e27183. 22073281 10.1371/journal.pone.0027183 PMC3206939

[39] Li DM, Sun H (1998) Pten/mmac1/tep1 suppresses the tumorigenicity and induces g1 cell cycle arrest in human glioblastoma cells. Proceedings of the National Academy of Sciences 95: 15406–15411. 10.1073/pnas.95.26.15406 PMC28055 9860981

[40] Liu Y, Shete S, Etzel CJ, Scheurer M, Alexiou G, et al (2010) Polymorphisms of lig4, btbd2, hmga2, and rtel1 genes involved in the double-strand break repair pathway predict glioblastoma survival. Journal of Clinical Oncology 28: 2467–2474. 20368557 10.1200/JCO.2009.26.6213 PMC2881725

[41] Watanabe A, Ogiwara H, Ehata S, Mukasa A, Ishikawa S, et al (2011) Homozygously deleted gene dach1 regulates tumor-initiating activity of glioma cells. Proceedings of the National Academy of Sciences 108: 12384–12389. 10.1073/pnas.0906930108 PMC3145721 21750150

[42] del Mar Inda M, Fan X, Muñoz J, Perot C, Fauvet D, et al (2003) Chromosomal abnormalities in human glioblastomas: Gain in chromosome 7p correlating with loss in chromosome 10q. Molecular Carcinogenesis 36: 6–14. 12503074 10.1002/mc.10085

[43] Henson J, Schnitker B, Correa K, von Deimling A, Fassbender F, et al (1994) The retinoblastoma gene is involved in malignant progression of astrocytomas. Ann Neurol 36: 714–21. 7979217 10.1002/ana.410360505

[44] Carrero P, Okamoto K, Coumailleau P, O'Brien S, Tanaka H, et al (2000) Redox-regulated recruitment of the transcriptional coactivators creb-binding protein and src-1 to hypoxia-inducible factor 1. Molecular and Cellular Biology 20: 402–415. 10594042 10.1128/mcb.20.1.402-415.2000 PMC85095

[45] Donovan L, Potter N, Warr T, Pilkington G (2012) A Prominin-1-Rich Pediatric Glioblastoma: Biologic Behavior Is Determined by Oxygen Tension-Modulated CD133 Expression but Not Accompanied by Underlying Molecular Profiles. Transl Oncol 5: 141–54. 22741033 10.1593/tlo.11337 PMC3384268

[46] Irminger-Finger I, Leung WC, Li J, Dubois-Dauphin M, Harb J, et al (2001) Identification of bard1 as mediator between proapoptotic stress and p53-dependent apoptosis. Molecular Cell 8: 1255–1266. 11779501 10.1016/s1097-2765(01)00406-3

[47] Sobottka S, Haase M, Fitze G, Hahn M, Schackert H, et al (2000) Frequent loss of heterozygosity at the 19p13.3 locus without lkb1/stk11 mutations in human carcinoma metastases to the brain. Journal of Neuro-Oncology 49: 187–195. 11212897 10.1023/a:1006442024874

[48] Kanu OO, Hughes B, Di C, Lin N, Fu J, et al (2009) Glioblastoma Multiforme Oncogenomics and Signaling Pathways. Clinical Medicine Insights: Oncology 3: 39. 10.4137/cmo.s1008 PMC2748278 19777070

[49] Chen H, Ye D, Xie X, Chen B, Lu W (2004) Vegf, vegfrs expressions and activated stats in ovarian epithelial carcinoma. Gynecologic Oncology 94: 630–635. 15350351 10.1016/j.ygyno.2004.05.056

[50] Rosenthal LA, Winestock KD, Finbloom DS (1997) Il-2 and il-7 induce heterodimerization of stat5 isoforms in human peripheral blood t lymphoblasts. Cellular Immunology 181: 172–181. 9398404 10.1006/cimm.1997.1208

[51] Hennighausen L, Robinson G (2005) Information networks in the mammary gland. Nat Rev Mol Cell Biol 6: 715–25. 16231422 10.1038/nrm1714

[52] Bouilly J, Sonigo C, Auffret J, Gibori G, Binart N (2012) Prolactin signaling mechanisms in ovary. Molecular and Cellular Endocrinology 356: 80–87. 21664429 10.1016/j.mce.2011.05.004

[53] Grandis JR, Drenning SD, Chakraborty A, Zhou MY, Zeng Q, et al (1998) Requirement of stat3 but not stat1 activation for epidermal growth factor receptor- mediated cell growth in vitro. The Journal of Clinical Investigation 102: 1385–1392. 9769331 10.1172/JCI3785 PMC508986

[54] Guren T, Abrahamsen H, Thoresen G, Babaie E, Berg T, et al (1999) EGF-induced activation of Stat1, Stat3, and Stat5b is unrelated to the stimulation of DNA synthesis in cultured hepatocytes. Biochem Biophys Res Commun 258: 565–71. 10329425 10.1006/bbrc.1999.0684

[55] Colomiere M, Ward A, Riley C, Trenerry M, Cameron-Smith D, et al (2009) Cross talk of signals between EGFR and IL-6R through JAK2/STAT3 mediate epithelial-mesenchymal transition in ovarian carcinomas. Br J Cancer 100: 134–44. 19088723 10.1038/sj.bjc.6604794 PMC2634691

[56] Benito M, Valverde A, Lorenzo M (1996) IGF-I: a mitogen also involved in differentiation processes in mammalian cells. Int J Biochem Cell Biol 28: 499–510. 8697095 10.1016/1357-2725(95)00168-9

[57] Ren Z, Schaefer T (2002) ErbB-2 activates Stat3 alpha in a Src- and JAK2-dependent manner. J Biol Chem 277: 38486–93. 11940572 10.1074/jbc.M112438200

[58] Jones R, Gordus A, Krall J, MacBeath G (2006) A quantitative protein interaction network for the ErbB receptors using protein microarrays. Nature 439: 168–74. 16273093 10.1038/nature04177

[59] Fujitani Y, Hibi M, Fukada T, Takahashi-Tezuka M, Yoshida H, et al (1997) An alternative pathway for STAT activation that is mediated by the direct interaction between JAK and STAT. Oncogene 14: 751–61. 9047382 10.1038/sj.onc.1200907

[60] Barahmand-Pour F, Meinke A, Groner B, Decker T (1998) Jak2-Stat5 interactions analyzed in yeast. J Biol Chem 273: 12567–75. 9575217 10.1074/jbc.273.20.12567

[61] Stortelers C, Souriau C, van Liempt E, van de Poll M, van Zoelen E (2002) Role of the N-terminus of epidermal growth factor in ErbB-2/ErbB-3 binding studied by phage display. Biochemistry 41: 8732–41. 12093292 10.1021/bi025878c

[62] Thornton C, Yaka R, Dinh S, Ron D (2003) H-Ras modulates N-methyl-D-aspartate receptor function via inhibition of Src tyrosine kinase activity. J Biol Chem 278: 23823–9. 12695509 10.1074/jbc.M302389200 PMC1196389

[63] Yip A, Horvath S (2007) Gene network interconnectedness and the generalized topological overlap measure. BMC Bioinformatics 8: 22. 17250769 10.1186/1471-2105-8-22 PMC1797055

[64] Langfelder P, Zhang B, Horvath S (2008) Defining clusters from a hierarchical cluster tree: the Dynamic Tree Cut package for R. Bioinformatics. 24: 719–720. 10.1093/bioinformatics/btm563 18024473

[65] Girvan M, Newman MEJ (2002) Community structure in social and biological networks. Proceedings of the National Academy of Sciences 99: 7821–7826. 10.1073/pnas.122653799 PMC122977 12060727

[66] Ulitsky I, Shamir R (2007) Identification of functional modules using network topology and highthroughput data. BMC Systems Biology 1: 8. 17408515 10.1186/1752-0509-1-8 PMC1839897

[67] Li M, Wu X, Wang J, Pan Y (2012) Towards the identification of protein complexes and functional modules by integrating ppi network and gene expression data. BMC Bioinformatics 13: 109. 22621308 10.1186/1471-2105-13-109 PMC3434013

[68] Nepusz T, Yu H, Paccanaro A (2012) Detecting overlapping protein complexes in protein-protein interaction networks. Nat Meth 9: 471–472. 10.1038/nmeth.1938 PMC3543700 22426491

[69] Keshava Prasad TS, Goel R, Kandasamy K, Keerthikumar S, Kumar S, et al (2009) Human protein reference database?2009 update. Nucleic Acids Research 37: D767–D772. 18988627 10.1093/nar/gkn892 PMC2686490

[70] Shannon P, Markiel A, Ozier O, Baliga NS, Wang JT, et al (2003) Cytoscape: A Software Environment for Integrated Models of Biomolecular Interaction Networks. Genome Research 13: 2498–2504. 14597658 10.1101/gr.1239303 PMC403769

