# Correction: Voting-Based Cancer Module Identification by Combining Topological and Data-Driven Properties

**DOI:** 10.1371/annotation/9a79fb7e-dce5-4525-9df5-dc130679c155

**Published:** 2014-01-30

**Authors:** A. K. M. Azad, Hyunju Lee

Errors occurred in several of the figures in this article.

In Figure 1, the orientation of one arrow is misdirected. Please see the corrected version of Figure 1 here: 

**Figure:**
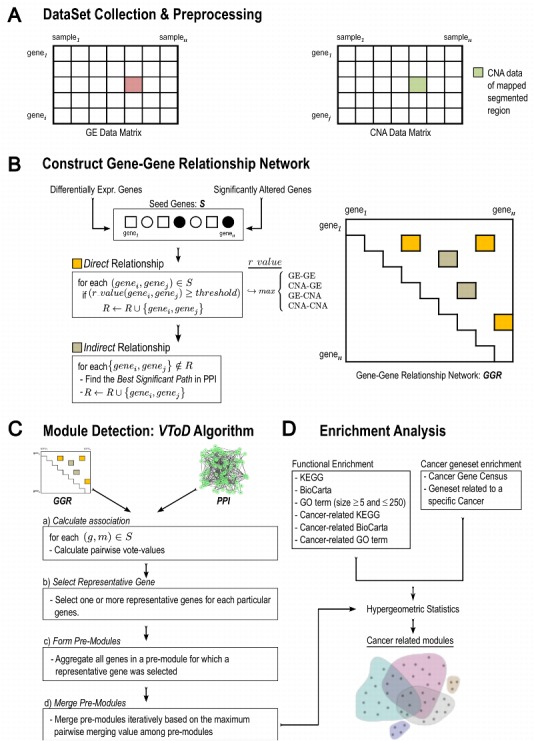


In Figure 2, some vital parts of the figure are missing. Please see the corrected version of Figure 2 here: 

**Figure:**
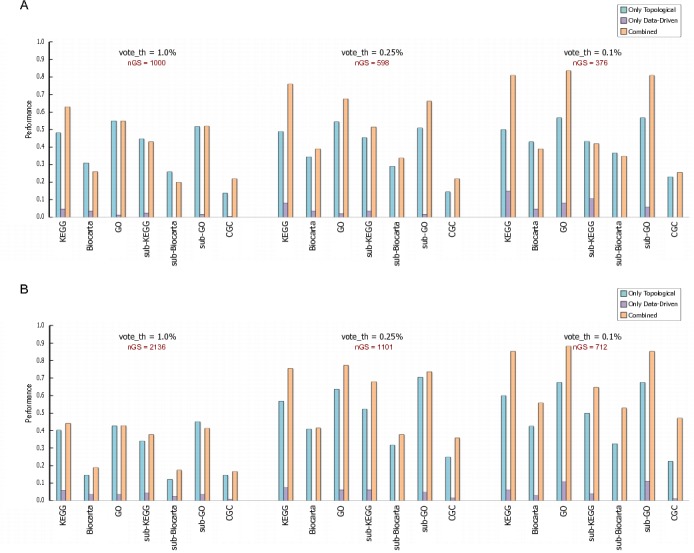


Figure S3 is the incorrect figure. Please see the correct Figure S3 here: 

Lastly, Figures S7 and S8 were interchanged.

