# Correction: Voting-Based Cancer Module Identification by Combining Topological and Data-Driven Properties

**DOI:** 10.1371/journal.pone.0096883

**Published:** 2014-04-28

**Authors:** The PLOS ONE Staff

There is an error in the Correction published on January 30, 2014. The correction reads that “Figures S7 and S8 were interchanged.” This is correct, however, Figure S8 is also incorrect and the correct file can be downloaded below.

## Supporting Information

Figure S8 **Distributions of topological and data-driven properties in merging pre-modules.** (A) and (C) are distributions of topological property values of all pairs of pre-modules, and (B) and (D) are distributions of data-driven property values of all pairs of pre-modules, for GBM and OVC, respectively.
(TIF)
