# Supplementary material for: Voting-Based Cancer Module Identification by Combining Topological and Data-Driven Properties
Source: PLoS One. 2013 Aug 5;8(8):e70498. doi: 10.1371/journal.pone.0070498 (PMC3734239; doi:10.1371/journal.pone.0070498)
Supplement: File S1 — Descriptions about finding indirect relationships, and topological and data-driven properties in merging pre-modules. (PDF) [file pone.0070498.s025.pdf]

Supplementary Methods

Finding indirect relationships

While constructing the network GGR, we first sought the direct relationships among the genes $\in S$ and then searched statistically significant indirect relationships among them for which direct relationships were not defined. To search indirect relationships, we had to consider the subnetwork GGR^{*}:= (S,R^{*}) as a fully-connected network where $R^{*} = \{(g_i, g_j)|(g_i, g_j) \notin R\}$. Thus, searching statistically significant pairwise indirect relationships considering a set of intermediate genes lead to a NP-hard problem. In order to solve this problem heuristically, we employed PPI information to restrict pairwise adjacency in GGR^{*} by converting our problem into a problem of finding statistically significant simple path between the genes in R^{*}. Also, since the length of such paths was also crucial in terms of time-complexity, we constrained our search with the path length = 2. In searching for indirect relationships, we greedily selected a path with the maximum average PPI connectivity and then assessed the statistical significance of the path by using random PPI newtworks which might yield the local optimum path. But, assessing all the enumerated paths and then choosing the one with maximum average PPI connectivity would be computationally expensive. However, this assessment was clearly assisted by hub genes in PPI graph which resolves the trade-off between avoiding possible local optimum results and the computational time-complexity.

Topological and data-driven property in merging pre-modules

At the each iteration of the pre-module merging algorithm, we selected two pre-modules that had the maximum merging value. The stopping criterion of this merging pre-modules was selected based on the statistical validation (see Methods section). Figures S3A and S3C show the change of the maximum merging values at each iteration of merging pre-modules until only two modules left, for GBM and OVC data sets, respectively. As both topological and data-driven properties were considered for calculating merging value between two pre-modules, we inspected their corresponding values at each merging step. Figure S9 shows that at each merging step both properties were significantly contributing to make the merging value maximum, and explains the importance of both topological and biological properties while merging pre-modules.
