# Supplementary figures and images for: Correction: Voting-Based Cancer Module Identification by Combining Topological and Data-Driven Properties
Source: PLoS One. 2014 Jan 30;9(1):10.1371/annotation/9a79fb7e-dce5-4525-9df5-dc130679c155. doi: 10.1371/annotation/9a79fb7e-dce5-4525-9df5-dc130679c155 (PMC3907584; doi:10.1371/annotation/9a79fb7e-dce5-4525-9df5-dc130679c155)

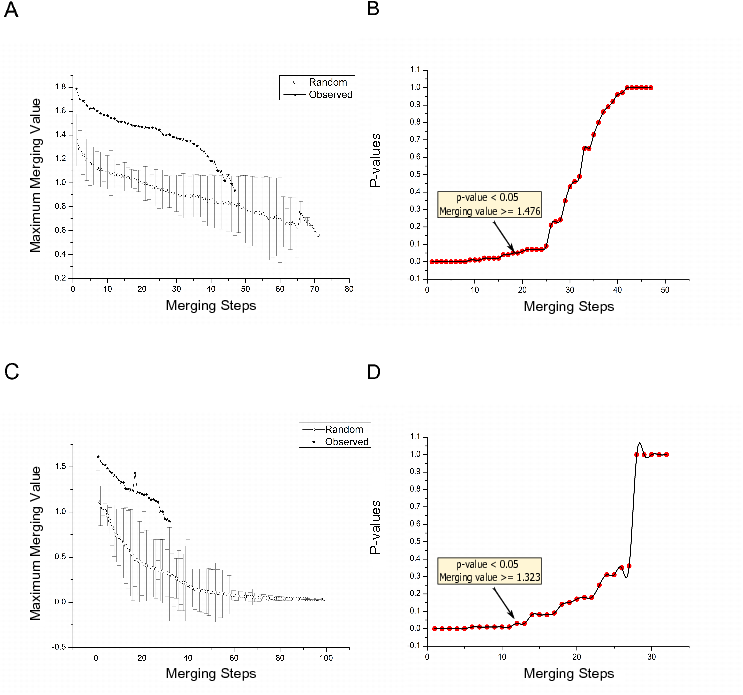

Supplement: Supplementary file 1 [file pone.9a79fb7e-dce5-4525-9df5-dc130679c155.s001.tif]
