# Supplementary figures and images for: Correction: Voting-Based Cancer Module Identification by Combining Topological and Data-Driven Properties
Source: PLoS One. 2014 Apr 28;9(4):e96883. doi: 10.1371/journal.pone.0096883 (PMC4002484; doi:10.1371/journal.pone.0096883)

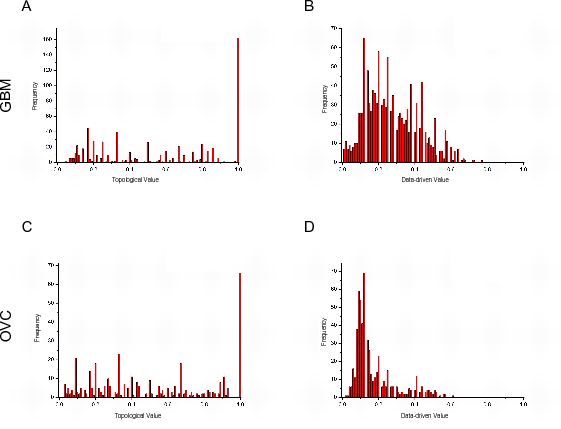

Supplement: Figure S8 — Distributions of topological and data-driven properties in merging pre-modules. (A) and (C) are distributions of topological property values of all pairs of pre-modules, and (B) and (D) are distributions of data-driven property values of all pairs of pre-modules, for GBM and OVC, respectively. (TIF) [file pone.0096883.s001.tif]
